# Aspects of Langerhans cells and TNF-α in the cutaneous immunity of anogenital warts^☆☆^^☆^

**DOI:** 10.1016/j.abd.2019.06.007

**Published:** 2020-02-12

**Authors:** John Verrinder Veasey, Adriana Bittencourt Campaner, Rute Facchini Lellis

**Affiliations:** a Dermatology Clinic, Santa Casa de Misericórdia de São Paulo, Vila Buarque, SP, Brazil; b Department of Ginecology and Obstetrics, Santa Casa de Misericórdia de São Paulo, Vila Buarque, SP, Brazil; c Pathology Laboratory, Santa Casa de Misericórdia de São Paulo, Vila Buarque, SP, Brazil

**Keywords:** Condylomata acuminata, Immunity, Langerhans cells, Tumor necrosis factor-alpha, Warts

## Abstract

**Background:**

Anogenital warts are the leading sexually transmitted infection in patients seeking care at specialized clinics. They may display a vast array of forms, according to the interaction of the virus with the host's immunity. Cellular immunity is the epithelium's main form of defense against the virus, involving an active participation of the Langerhans cells and pro-inflammatory cytokines such as TNF-α.

**Objective:**

To assess the epithelial immune response of anogenital warts in males, according to the number of lesions presented.

**Methods:**

This is a prospective, cross-sectional study carried out at the dermatology outpatient clinic in a tertiary hospital. We included male patients over 18 years of age without comorbidities who had anogenital condylomata and no previous treatments.In order to evaluate the local epithelial immunity, the lesions were quantified, then removed and employed in CD1a immunohistochemistry assays for assessing the morphometry and morphology of Langerhans cells; TNF-α; reaction was used for determining cytokine positivity in the epithelium.

**Results:**

48 patients were included in the study. There was no statistically significant difference as to the number of Langerhans cells, in their morphology, or the presence of TNF-α. However, patients presenting with more Langerhans cells in the lesions had cells with a star-like and dendritic morphology, whereas in those with a lower cell count had cells with a rounded morphology and no dendrites (*p* < 0.001).

**Study limitations:**

Small number of patients analyzed.

**Conclusion:**

There was no difference in epithelial immunity between patients having few or many anogenital condyloma lesions as measured by the morphology and morphometry of Langerhans cells and TNF-α; positivity. Such an assessment employing immunity markers differing from the usual ones is expected to yield useful results.

## Introduction

Anogenital warts, also known as condylomata acuminata (CA), are sexually transmitted infections (STIs) caused by the human papillomavirus (HPV). They are considered the most frequent STI in developing countries and can now be classified as a global epidemic. Anogenital warts can be caused by several HPV serotypes and are most often caused by low-risk HPV types 6 and 11.^1^ They can cause psychological distress by making patients feel ashamed and less attractive, thereby reducing their quality of life. High levels of anxiety, anger, and depression have been shown to be linked to the diagnosis and treatment of genital warts, and approximately two-thirds of the patients make lifestyle changes in relation to sexual intercourse. In addition, sexual pleasure and activity are negatively affected as well.^2^

The progression of HPV-induced lesions is correlated with viral type, genetic factors, environmental factors, and mostly with host immunity.^3^, ^4^ This immune defense of the epithelium has the participation of dendritic cells, which, in the skin, are represented by the Langerhans cells (LC) and keratinocytes themselves.^5^ As the primary target of HPV, keratinocytes play an important role during the onset of virus infection and subsequently becomes involved in promoting an effective adaptive immune response. Keratinocytes act as antigen-presenting cells and are capable of inducing the expression of cytokines: they express Toll-like receptors (TLRs), which in recognizing pathogen-associated molecular patterns (PAMPs), are themselves activated and thus initiate signaling pathways that result in innate and adaptive immune responses.^6^ The activation of these receptors, in turn, promotes the production of cytokines, thereby creating a powerful pro-inflammatory environment, in particular upon the activation of TLR-9, which stimulates keratinocyte to produce TNF-α.^6^, ^7^, ^8^

Langerhans cells are dendritic cells usually found in the spinous layer of the epidermis, whose distribution through the human body ranges from 200 cells/mm^2^ in the palmoplantar and genital region to about 1000 mm^2^ in head, neck, trunk, and limbs.^1^ They play an important role in skin immune response in as much as they can capture and present antigens to T lymphocytes. LC maturation also plays a key role in efficiently inducing T-cell responses, with such maturation signals, in turn, originating from the LC communicating with the surrounding tissue through proinflammatory tissue-derived cytokines, such as TNF-α.^8^, ^9^

Nevertheless, HPV can modulate LC adhesion and migration and may also affect the LC count present in the infected epithelium. Morelli et al.^10^ found that LC were significantly decreased in the genital tract infected by HPV in males and females, whereas Banchereau et al.^11^ also reported abnormal morphology and LC distribution in condyloma acuminata lesions.^5^ We believe that the number of condylomatous lesions that patients develop could correlate with the presence of effective immunity. To date, there are few related studies that assess such correlation.

Our study aims to assess the epithelial immunity of anogenital warts of men treated at a dermatology outpatient clinic, correlate the clinical presentation of warts (number of lesions) with local immunity, as measured by the quantification (morphometry) and morphological aspects of the Langerhans cells in them, and additionally correlate the number of lesions with either the presence or absence of TNF-α labeling in them.

## Materials and methods

We conducted a prospective cross-sectional study at the Outpatient Clinic of Sexually Transmitted Infections at the Dermatology Clinic in a Tertiary Hospital located in the city of São Paulo – Brazil from January 2015 to December 2017. The research project was accepted by the institution's research ethics committee (CAAE no. 40848115.4.0000.5479).

The sample size was calculated at a 5% significance level, with a test power of 80%, based on a formula calculated in comparison of proportions in which the calculation inputs came from the results obtained in a pilot study with 24 patients. From this calculation, a number of 48 patients was reached.

Included in this study were male patients, aged 18 years and older, receiving treatment at the outpatient clinic, presenting with anogenital warts located anywhere and agreeing to participate in the study, having signed the voluntary informed consent form (VICF). They were included regardless of the number of warts and their size. Still, the warts should have appeared more than one month previously to them being included in the study. Excluded from the study were: systemically immunocompromised patients (transplanted patients; patients testing seropositive for the human immunodeficiency virus (HIV); having acquired immunodeficiency syndrome (AIDS); and cancer patients); patients on systemic immunosuppressive medications (corticoid therapy, immunotherapy, chemotherapy); patients who had received any previous or current topical treatment for the lesion (either involving medications or not); patients presenting with lesions that could not undergo surgical excision.

All patients who accepted to participate in the study were assessed during a clinical anamnesis, during which the number and site of their condylomata were determined. The lesions were removed in their entirety by surgical excision the lesions. Patients with more than one lesion underwent excision of all condylomata in one operative procedure. For all paraffin blocks containing samples from the condylomata, two immunohistochemical reactions were performed: one with the CD1a antibody to assess Langerhans cells and another with TNF-α to test the reaction positivity in the epidermis.

**Figure 1:**
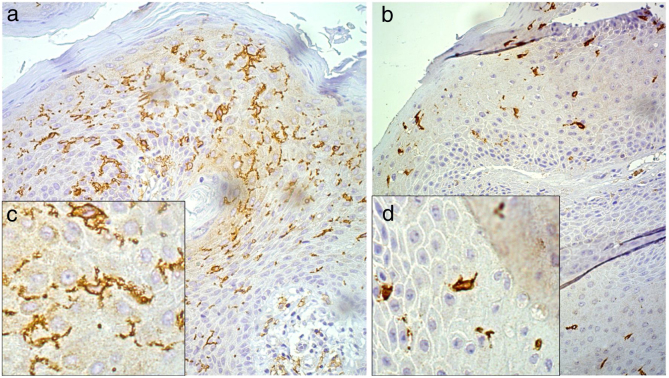
Morphology of Langerhans cells as evidenced by CD1a immunohistochemistry assays, under 200× magnification (A and B) and 32,000× digital zoom (C and D), with Langerhans cells stained in brown. A and C, cells with evidenced dendritic projections. B and D, rounded cells with few or no dendritic projections.

Following the immunohistochemistry reactions for CD1a, the slides were used in the Langerhans cell (LC) morphometry assays by employing the technique described by Campaner and Galvão.^12^ LC morphometry was obtained at a 200× magnification with the aid of the Neubauer chamber, counting 10 random fields of each slide, and its result was quantified per square millimeter. For the LC morphology analysis, the cells were evaluated if they displayed a star-like appearance with long, well-defined dendrites, or a rounded appearance, without dendrites or with but short dendrites (Fig. 1).

In the immunohistochemistry assays for TNF-α, the samples that developed positive reaction into the cytoplasmic membrane of epidermal cells were considered positive, according to the standards shown in the reagent's manual provided by its manufacturer, and negative if they did not display such reactions.

Comparisons were made between number of lesions, number of Langerhans cells, Langerhans cell morphology, and TNF-α analysis. With respect to the number of lesions, the patients were classified into two groups: those having up to five lesions, and those having over five lesions.

## Results

During the study period, 492 patients with a clinical suspicion of anogenital condyloma acuminata were treated. Of these patients, 443 were excluded from the study due to the following reasons: they were female; they were under 18 years of age; they had associated immunosuppression (such as HIV infection, or transplants); they had undergone previous treatment(s) for the referred lesion(s); or because their diagnosis of anogenital condyloma had not been confirmed. This left 49 patients who met the inclusion/exclusion criteria. One patient was subsequently excluded following the immunohistochemical tests since the collected material was not representative (insufficient material). Thus, the final number of patients assessed in the study was 48, a number compatible with the aforementioned sample size calculation.

Among the 48 patients evaluated, the lesions predominantly affected the extragenital sites, totaling 47 cases (61.9%), followed by genital sites in 29 cases (38.1%); 27 of the 48 patients (56.3%) had warts at only one site, whereas 21 patients (43.7%) had lesions at several sites (Table 1).

**Table 1:** Site of condylomata lesions removed from the 48 patients.

Lesions location	*N*	%
*Genital*		
Penis body	19	25
Scrotum	4	5.3
Inner foreskin	2	2.6
Balano-prepucial groove	2	2.6
Glans	2	2.6

*Extragenital*		
Pubis	21	27.7
Anal	12	15.8
Inguinal	10	13.1
Perineum	4	5.3

*Total*	76	100

*Number of affected sites*		
1 location	27	56.3
2 locations	17	35.5
3 locations	3	6.2
6 locations	1	2

*Total*	48	100

With respect to the number of lesions, the patients had a number ranging from one to 20, with an average of 8.4 ± 7.5 lesions, and a median of 4.5. These data are shown in table 2. The distribution of the number of lesions varied, which did not allow for representativeness with a satisfactory sample of patients in each of the groups, as defined by the number of lesions. The 48 patients were distributed among 17 groups with a number of lesions ranging from 1 to 20. This fact does not permit a valid correlation to each variable of the study, such as the number of Langerhans cell and TNF-α reaction for instance. In view of this situation, the 48 patients were divided according to their number of lesions into two groups: patients with up to five lesions, and patients with over five lesions. The first group was comprised of 28 patients (58.3%), whereas the second group, 20 (41.7%). This division into two groups allowed for more equivalent representativeness of the 48 patients in the study, making it more appropriate to compare the number of lesions with the other factors studied. The number of five lesions to divide the groups was chosen against the practical applicability of the therapeutic approach used in the treatment of anogenital condylomata: in the dermatological practice, we usually employ destructive procedures, such as surgical exeresis or electrocauterization of the lesions with infiltrative anesthesia, in the cases involving fewer lesions, and non-destructive procedures, such as the use of topical substances (e.g. podophyllin, imiquimod, 5-fluorouracil), to reduce lesions and destructive therapies at a later stage, in the case of patients with several of lesions.^13^

**Table 2:** Distribution of the number of condylomatous lesions among the 48 patients in the study.

N of lesions	N of patients	%
1	1	14.6
2	7	14.6
3	3	6.3
4	7	14.6
5	4	8.3
9	2	4.2
10	2	4.2
13	2	4.2
14	2	4.2
15	1	2.1
16	1	2.1
18	1	2.1
19	1	2.1
20	3	6.3
21	3	6.3
22	1	2.1
24	1	2.1

Total	48	100.0

The Langerhans cells’ morphometry results ranged from 18 to 245 cells/mm^2^, with a mean of 138.75 ± 65.16 cells, and median of 196 cells/mm^2^. With regard to the Langerhans cells’ morphology, only two types of presentations were observed: star-like cells with thin and long dendrites, or rounded cells with short and/or absent dendrites (Fig. 1). Of the 48 patients studied, 22 (45.8%) had rounded Langerhans cells, whereas 27 (56.3%) had star-like cells. TNF-α was positive in 27 (56.3%) and negative in 21 (43.8%) of the 48 samples.

In comparing the number of Langerhans cells per square millimeter with the patient's number of lesions, no statistically significant difference was found between the groups (*t*-test, *p* = 0.784). The comparison between the Langerhans cells’ morphology and the number of lesions observed in the two groups did not result in a statistically significant difference either (Chi-squared test, *p* = 0.922). A correlation analysis between TNF-α positivity was also carried out in both groups, i.e. the one group of patients having few lesions and the other group of patients with many lesions. In this comparison, no statistically significant result was observed (Chi-squared test, *p* = 0.302).

When correlating Langerhans cells’ morphology and morphometry, a larger presence of star-like cells with dendrites was found in the lesions with a higher number of cells, whereas a smaller presence of oval Langerhans cells with few or no dendrites was found in the lesions with a lower number of cells. The comparison between these two groups showed a statistically significant change, with *p* < 0.001 as assessed by the Mann–Whitney test (Fig. 1).

## Discussion

According to the World Health Organization (WHO), in developing countries, sexually transmitted infections and their complications are among the top five categories of diseases for which adults seek health care. In the United States, anogenital condyloma is thought to affect about 1% of the population, with 500,000 new cases per year and 300,000 medical consultations per year.^4^ Regarding Brazil, WHO estimate an annual occurrence of 684,400 new cases.^14^ Fagundes et al. conducted an epidemiological study in Brazil at an STI reference clinic in Brazil in which 4128 patients received treatment over a 10 year period.^15^ Of these patients, the majority were males (76%) and the leading disease diagnosed was condyloma acuminate (29%). Another finding of the study was that 7.9% of the individuals were diagnosed with HIV seropositivity; of these, 90.8% were males, which yields a M:F ratio of 9.9:1.^15^

HPV parasitism at the anogenital site of males can lead to the appearance of condylomata; cancer precursor lesions; and aggressive, mutilating and fatal neoplasms.^3^, ^14^, ^16^ Among these lesions, cancer and its precursor lesions are rare, with an increase in the incidence of anal cancer being found mainly in Men who have Sex with Men (MSM).^17^ Although condylomata are benign lesions, they have a considerable incidence in the population with psychological repercussions on the patient, which can be devastating in several psycho-social fields.^2^ Hence, a better understanding of the immunology of HPV infection would be extremely valuable in the therapeutic and prognostic programming of the lesions.

The immune response to HPV is known to be based on the recognition by dendritic cells present in the epidermis, represented by Langerhans cells. They act as antigen-presenting cells (APCs) that absorb and process the epicutaneous antigen, migrate to the regional lymph nodes, and then present the processed antigen to virgin T-cells, thereby initiating primary immune responses.^7^, ^9^, ^18^ These T-cells, sensitized due to antigen presentation, return to the infected epithelium, fighting viral infection by producing cytokines. Even though LC migration is well known to be required for antigen presentation, little is known about the molecular mechanisms involved. Studies indicate that pro-inflammatory cytokines such as IL-1 and TNF-α stimulate this migration, whereas cytokines such as IL-10 inhibit it.^6^, ^7^, ^8^, ^9^, ^18^

In our study, we attempted to correlate the number of lesions with the local immunity, postulating that the larger the number of lesions a patient has, the worse their immune response would be. Evidence on the correlation between condyloma acuminata and local immunity as assessed by morphometry and LC morphology has already been reported elsewhere, indicating that patients with lesions have a smaller LC counts when compared to patients without condylomata.^5^ Feng et al. investigated LC morphometry in condyloma lesions by CD1a immunohistochemical tests and found a statistically smaller number (26.31 ± 18.84) if compared to healthy skill controls (72.00 ± 27.20) (*p* < 0.001).^5^ In that same study, its authors demonstrated that Birbeck granules were reduced in LC from lesions by using electron microscopy as compared to normal controls, thus corresponding to degenerative LC alterations in condylomata.^5^ In an earlier study by Mc Ardle et al., tissue LC was immunohistochemically quantified with S100. Patients with condylomata were shown to have a reduced cell count compared to normal epithelium.^19^ We believe that there are two possibilities for interpreting the lower LC count in the condylomatous lesions as compared to healthy skin: either because migration to the lymph node had already taken place at the time of biopsy, or because LC had been suppressed by the viral infection. No studies were found in the literature comparing LC in patients having many and few condyloma lesions, as was done in the current study.

In our study, the number of warts correlated with LC count and TNF-α tissue marking, and we observed no statistically significant differences between the morphology and morphometry of LC in the groups of patients with either few or many lesions. These findings were contrary to what we had expected, since we believed that the higher the number of lesions, the lower the number of cells. TNF-α analysis was performed in order to evaluate if LC morphometry would correlate with this cytokine, capable of stimulation cell migration. However, no correlation was observed between TNF-α labeling and the morphometry or morphology of CL, preventing us from making any further statements about the interaction of these factors.

A relevant positive finding in our study, nonetheless, was the presence of a correlation between LC count and their morphology. Samples with a higher number of Langerhans cells had star-like cells with long, multiple and evident dendrites, whereas samples with lower LC counts had rounded cells with few or no dendrites at all. In the authors’ interpretation, this finding seems to indicate that the epithelium sensitized with the immunological onset of antigen presentation to LC presents a smaller number of cells due to the probable migration to the lymph node, and few cells still present in the tissue are already retracted and prepared to sequence in the cascade immunology that is to come, whereas in the epithelium, with many star-like Langerhans cells, the sensitization of LC, with their morphological alteration and migration to the lymph node, has not yet begun.

## Conclusion

In conclusion, we believe that the epithelial immunity of anogenital warts is a complex and multifactorial process. The lack of statistical correlation between the investigated LC traits and the number of lesions in the patient raises the possibility that there can be subgroups divided according other variables with a statistically significant correlation. This fact motivated the authors to include in this study the TNF-α analysis together with the variables for evaluating the epithelial immunity in the condylomata. Yet, that did not correlate with the indexes we analyzed. We believe that further studies on the epithelial immunity of anogenital condylomas should be performed in order to better elucidate the immunological aspects involved, and hence better understand their etiopathogeny and broaden the therapeutic possibilities for treating the lesions.

## Financial support

None declared.

## Authors’ contributions

John Verrinder Veasey: Statistic analysis; approval of the final version of the manuscript; conception and planning of the study; elaboration and writing of the manuscript; obtaining, analysis, and interpretation of the data; effective participation in research orientation; intellectual participation in the propaedeutic and/or therapeutic conduct of the studied cases; critical review of the literature; critical review of the manuscript.

Adriana Bittencourt Campaner: Approval of the final version of the manuscript; conception and planning of the study; elaboration and writing of the manuscript; obtaining, analysis, and interpretation of the data; effective participation in research orientation; critical review of the literature; critical review of the manuscript.

Rute Facchini Lellis: Obtaining, analysis, and interpretation of the data.

## Conflicts of interest

None declared.
